# Does the impact of medical publications vary by disease indication and publication type? An exploration using a novel, value-based, publication metric framework: the EMPIRE Index

**DOI:** 10.12688/f1000research.75805.3

**Published:** 2023-03-10

**Authors:** Tomas Rees, Avishek Pal

**Affiliations:** 1 Oxford PharmaGenesis, Oxford, Oxfordshire, OX13 5QJ, UK; 2 Novartis Pharma AG, Basel, Switzerland

**Keywords:** Altmetrics, bibliometrics, publication impact

## Abstract

**Background:** The EMPIRE (EMpirical Publication Impact and Reach Evaluation) Index is a value-based, multi-component metric framework to assess the impact of medical publications in terms of relevance to different stakeholders. It comprises three component scores (social, scholarly and societal impact), each incorporating related article-level metrics that indicate a different aspect of engagement with the publication. Here, we present an exploratory investigation of whether publication types or disease indications influence EMPIRE Index scores.

**Methods:** Article-level metrics were extracted and EMPIRE Index scores were calculated for 5825 journal articles published from 1 May 2017 to 1 May 2018, representing 12 disease indications (chosen to reflect a wide variety of common and rare diseases with a variety of aetiologies) and five publication types.

**Results:** There were significant differences in scores between article types and disease indications. Median (95% CI) social and scholarly impact scores ranged from 1.2 (0.3–1.6) to 4.8 (3.1–6.6), respectively, for phase 3 clinical trials, and from 0.3 (0.3–0.4) to 2.3 (1.9–2.6), respectively, for observational studies. Social and scholarly impact scores were highest for multiple sclerosis publications and lowest for non-small cell lung cancer publications. Systematic reviews achieved greater impact than regular reviews. Median trends in the social impact of different disease areas matched the level of public interest as assessed through Google search interest. Although most articles did not register societal impact, mean societal impact scores were highest for migraine publications.

**Conclusions:** The EMPIRE Index successfully identified differences in impact by disease area and publication type, which supports the notion that the impact of each publication needs to be evaluated in the context of these factors, and potentially others. These findings should be considered when using the EMPIRE Index to assess publication impact.

## Introduction

**Figure 1.**
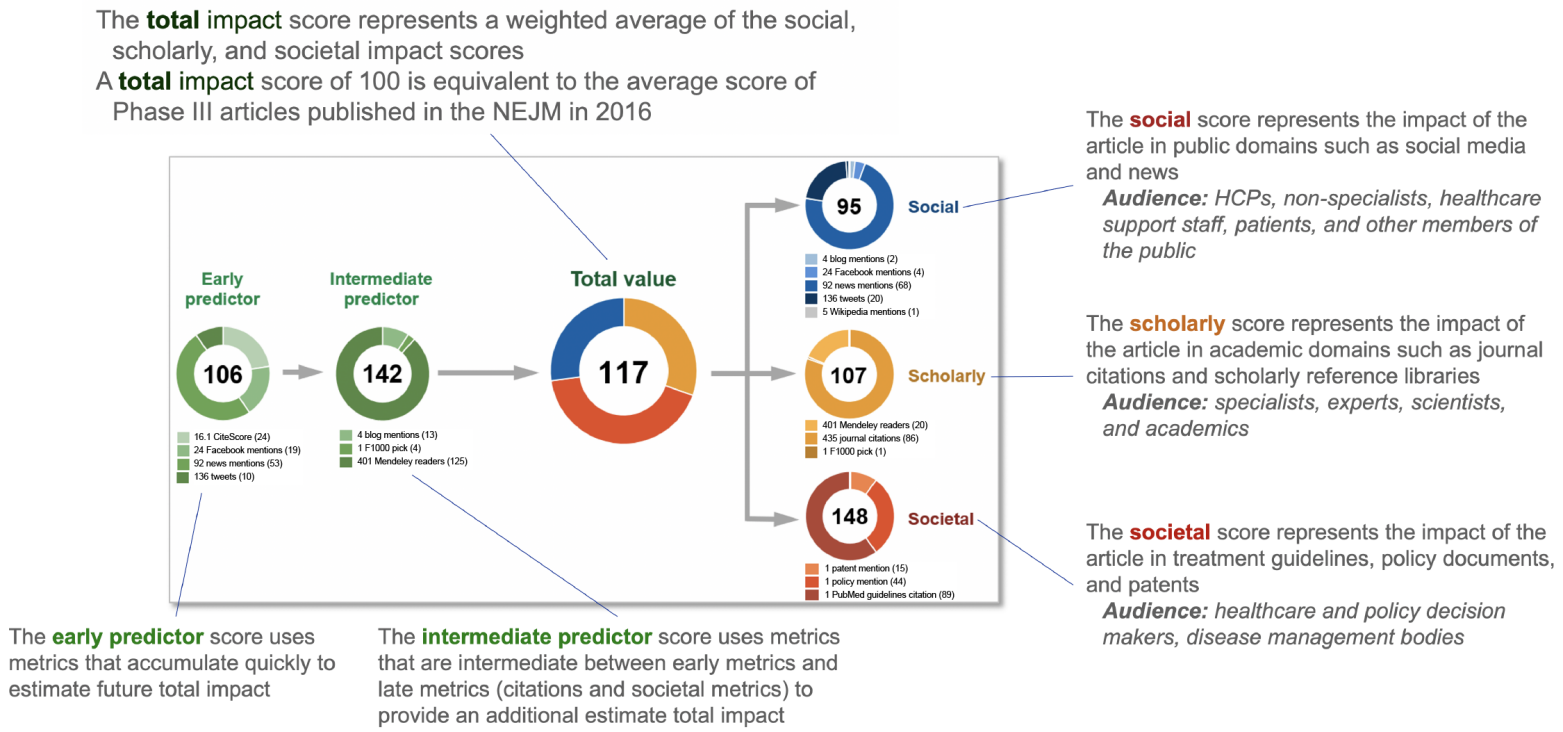
Example of the EMPIRE Index score for a single publication ^3^. HCP, healthcare provider; NEJM, *New England Journal of Medicine*.

Article-level measures of publication impact (ALMs, which include alternative metrics or altmetrics) can help to inform the impact of a publication among different audiences and in different contexts. Although the journal impact factor (JIF) and related scores such as CiteScore may help to identify journals with a high readership, they are widely recognised as being a poor indicator of the quality or impact of individual research articles ^1, 2^. We have previously described a novel approach to summarising ALMs, the EMPIRE (EMpirical Publication Impact and Reach Evaluation) Index, which uses article-level metrics to assess the impact of medical publications in terms relevant to different stakeholders ^3^. The EMPIRE Index provides component scores for scholarly, social and societal impact, as well as a total impact score and predictive reach metrics ( Figure 1). The scholarly component correlates weakly with CiteScore while the social score correlates closely with the Altmetric Attention Score, an altmetric-based score that is mostly related to citations in news and social media. The societal component, comprised of citations in guidelines, policy documents and patents, represents a distinct score.

The EMPIRE Index uses randomized controlled clinical trials published in the NEJM as benchmark, which studies that are selected by the journal editors as likely to be of the highest impact for the practice of medicine. However, this standard cannot be uniformly applied to all types of studies, across different disease areas and development stages of a therapy. It is widely recognised that publication metrics vary by discipline, and to facilitate the comparison of publication impact across different fields, field-normalised citation impacts are frequently calculated ^4^. Although evidence is limited, some research suggests that ALMs can also vary by publication type. For example, review articles in pharmacology journals can receive twice as many citations as original articles ^5^.

Therefore, the nuances of each field and publication type should be considered in order to understand what constitutes a typical score. This additional context is an important consideration as it allows users to compare scores with a relevant frame of reference and so to accurately interpret and utilise publication metrics and to derive meaningful insights.

It is likely that EMPIRE Index scores vary by disease area and by publication type. However, the scale and nature of these variations are unknown, which complicates efforts to compare scores of individual publications. This in turn limits the utility of the scale to identify ‘high impact’ publications, since what counts as an atypically high score will vary across therapy areas publication types.

In this brief report we present an exploratory investigation of the interaction between disease indications and publication types and typical EMPIRE Index scores. We sought to explore the variations that may exist in typical (i.e. average and median) EMPIRE Index scores across different therapy areas and publication types, and to explore the magnitude of these variations. This will allow the metrics of individual publications to be placed in a richer context of potentially comparable publications to facilitate interpretation of individual publication scores.

We also sought to provide additional context for the observed typical scores in two ways. We look to see if there are similar variations in journal CitesScore to explore whether variations could be expected based on differences in this journal-level indicator. We also look to see if there are similar variations in public interest in therapy areas we explore, with particular interest in comparing this with the EMPIRE Index includes a social score. For this we examine Google search trends, which have previously been used as a population-level measure of interest in health topics ^6^.

## Methods

This exploratory study investigated 12 disease indications, purposefully chosen to reflect a variety of common and rare diseases with a variety of aetiologies. Six of these were rare diseases, selected as a convenience sample of disease indications with which the authors were most familiar. No formal statistical power analysis was undertaken. However, we aimed for disease samples from approximately 1000 publications, which would enable publication type sub-analyses. The six rare disease samples were, therefore, pooled.

Relevant publications were identified for each disease by the appearance of the disease name in the publication title. We limited the search period to items with publication dates between 1 May 2017 and 1 May 2018, to give sufficient time for metrics to accumulate while also minimising the time-dependent variation in metrics.

The searches were conducted on PubMed between 22 June 2020 and 3 July 2020, using the following search string:

"(2017/05/01"[Date - Publication] : "2018/05/01"[Date - Publication]) AND "<disease name>"[TI]

For each disease, we conducted secondary searches for each publication type using PubMed tags for those of interest (i.e. the search string above and either “review”, "systematic review", "clinical trial, phase iii", “clinical trial” or "observational study"). PubMed publication types are metadata supplied by PubMed and derive originally from publisher submissions ^7^. PubMed IDs were entered into the Altmetric Explorer and PlumX dashboards and ALMs downloaded over the period 23 June 2020 to 11 July 2020. ALMs were assumed to be zero for any publication for which Altmetric Explorer did not return a result. We also obtained the journal CiteScore for all publications ^8^.

EMPIRE Index scores were calculated for all publications as described previously ^3^. Briefly, selected ALMs that compose the EMPIRE Index were weighted and aggregated to form three component scores (social impact, scholarly impact and societal impact), which were then summed to form a total impact score.

Each disease area comprised a different mixture of publication types, which we expected could confound the analysis; multivariate analysis on such a heterogenous, non-normal and zero-inflated data set is problematic. Therefore, we opted to create standardised samples through random polling.

A sample was created for each disease area with a standardised mix of publication types chosen to maximise the total number of publications retained (the standardised publication types [SPT] set). First, the two least common publication types (phase 3 clinical trials and systematic reviews) were excluded because of the high variation between disease areas and because they are largely subsets of other publication types (clinical trials and reviews, respectively). Although the observational studies publication type was only slightly more common than systematic reviews, it was retained as it was considered to be functionally very different from clinical trials and reviews. The proportions of each of the remaining three publication types were calculated for each disease set, as well as for the overall set. Publications were then trimmed from each disease set by random sampling, as needed, to match the proportions in the overall set. The trimmed publication sets formed the SPT set.

Similarly, each publication type comprised a different mix of diseases. A standardised disease areas (SDA) set was created by random sampling using a similar approach that ensured each publication type included the same mix of diseases, while maximising the total number of publications retained.

We downloaded weekly Google Trends data on relative interest over time for the period of interest for these diseases (May 1 2017 to May 1 2018). A score of 100 indicates the maximum interest in any week over the search period and across any of the search terms of interest. The year averages presented here are expressed relative to that maximum score.

As these analyses were exploratory, we primarily provide descriptive statistics and only minimal statistical analysis was undertaken. Intra-group differences were assessed using Kruskal-Wallis one-way analysis of variance, a non-parametric test for equality of population means (a significant result indicates that that at least one population median of one group is different from the population median of at least one other group).

## Results

### Sample characteristics

In total, 20 577 publications were identified across the 12 disease areas ^9^, of which 5825 (28%) were tagged with one of the publication types of interest ( Table 1). Table 1 also shows the Google search interest for each of these diseases.

**Table 1.** Numbers of publications identified in the search. Google search interest is the average weekly interest across the search period, and is a relative score 0–100 where 100 is the maximum score for any disease in any individual week.

	All publications	Clinical trial		Phase 3 clinical trial		Observational study		Review		Systematic review		Any identifiable publication type		Google search interest
	n	n		n		n		n		n		n		
Asthma	3487	225	6%	21	1%	114	3%	567	16%	100	3%	1027	29%	62
Migraine	996	88	9%	6	1%	36	4%	177	18%	29	3%	336	34%	88
MS	2986	175	6%	10	0%	80	3%	548	18%	78	3%	891	30%	72
NSCLC	3847	287	7%	57	1%	42	1%	417	11%	36	1%	839	22%	3
Psoriasis	1654	139	8%	53	3%	57	3%	281	17%	54	3%	584	35%	42
T2D	5341	644	12%	66	1%	213	4%	674	13%	187	4%	1784	33%	45
Rare diseases	2266	82	4%	18	1%	11	0%	243	11%	10	0%	364	16%	3
DLBLC	597	41	7%	10	2%	5	1%	45	8%	5	1%	106	18%	2
NASH	351	9	3%	0	0%	1	0%	64	18%	1	0%	75	21%	6
NET	187	3	2%	1	1%	1	1%	20	11%	1	1%	26	14%	3
SMA	196	8	4%	2	1%	0	0%	42	21%	0	0%	52	27%	4
TNBC	779	18	2%	4	1%	2	0%	60	8%	3	0%	87	11%	1
TSC	156	3	2%	1	1%	2	1%	12	8%	0	0%	18	12%	3
Total	**20 577**	**1640**	8%	**231**	1%	**553**	3%	**2907**	14%	**494**	2%	**5825**	28%	

DLBLC, diffuse large B-cell lymphoma; MS, multiple sclerosis; NASH, non-alcoholic steatohepatitis; NET, neuroendocrine tumour; NSCLC, non-small cell lung cancer; SMA, spinal muscular atrophy; T2D, type 2 diabetes; TNBC, triple-negative breast cancer; TSC, tuberous sclerosis complex.

### Analysis by publication type

The numbers of publications retained in the SDA set used for publication type comparisons (i.e. with the same disease indication composition for each publication type) are shown in Table 2.

**Table 2.** Numbers of publications retained in the SDA set used for publication type comparisons.

	Clinical trial	Phase 3 clinical trial	Observational study	Review	Systematic review	Proportion of total
	n	n	n	n	n	%
Asthma	197	11	51	399	44	19%
Migraine	66	4	17	133	15	6%
MS	175	10	45	354	39	17%
NSCLC	163	9	42	328	36	16%
Psoriasis	104	6	27	210	23	10%
T2D	334	19	86	674	74	32%
Total	1039	59	268	2098	231	100%

MS, multiple sclerosis; NSCLC, non-small cell lung cancer; T2D, type 2 diabetes.

**Figure 2.**
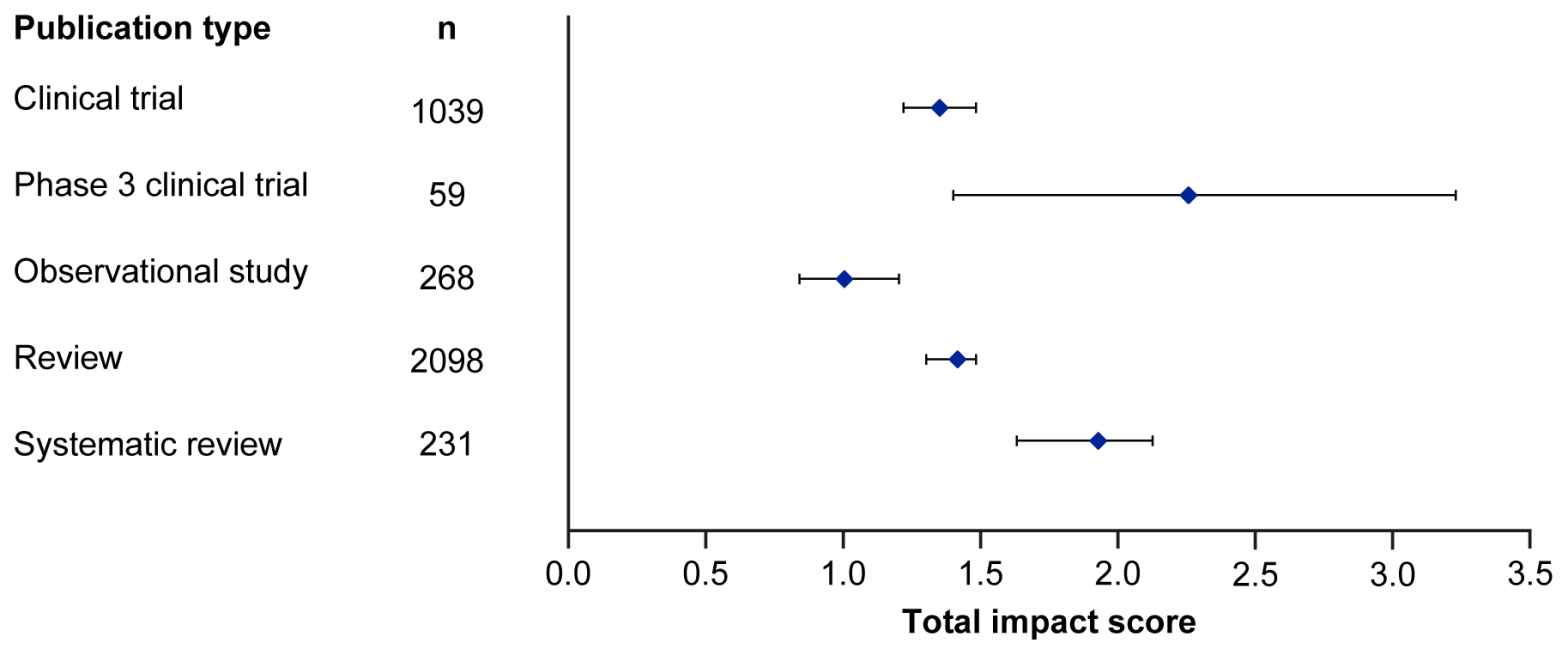
Median (95% CI) total impact scores for each publication type (standardised set). CI, confidence interval.

**Figure 3.**
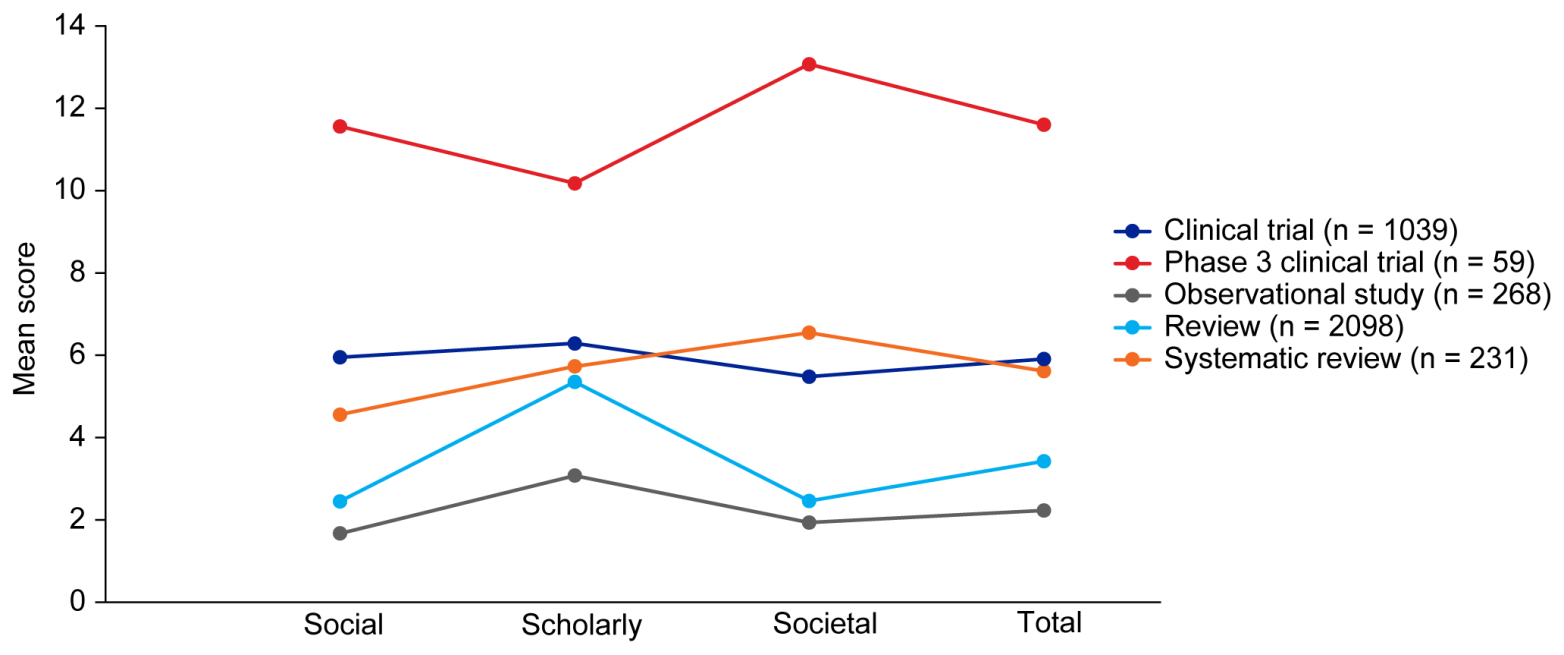
Mean EMPIRE Index scores for each publication type (SDA set). The interactive version (online only, accessible here: https://s3.eu-west-2.amazonaws.com/ox.em/webflow/p29ieu21/chart1.html) also shows mean EMPIRE Index scores for each disease by publication type (full set).

Median EMPIRE Index scores and CiteScores for each disease in the SDA set are shown in Figure 2 and Table 3. Mean EMPIRE Index scores, shown in Figure 3, broadly reflect the median scores. Statistical analysis indicated that there was some significant variation in the medians of each component as well as the total impact score and journal CiteScore. In general, the ranking of publication type is relatively consistent across different types of impact. Notably, phase 3 clinical trials had the highest median and mean scores, while observational studies had the lowest. Systematic reviews had higher impact than reviews. Most articles across all publication types had no societal impact, and significant differences in societal impact were driven by outliers. Of note, eight of the ten publications with the highest societal impact were clinical trials, and six of those were in non-small cell lung cancer (NSCLC).

**Table 3.** Median and maximum scores for each EMPIRE Index component and CiteScore by publication type (SDA set).

		Social			Scholarly			Societal			Total			CiteScore		
	n	Median	95% CI	Max	Median	95% CI	Max	Median	95% CI	Max	Median	95% CI	Max	Median	95% CI	Max
Clinical trial	1039	0.4	0.4–0.6	411.9	3.0	2.8–3.2	303.8	0.0	0.0–0.0	267.0	1.4	1.2–1.5	274.4	2.9	2.9–2.9	19.1
Phase 3 clinical trial	59	1.2	0.3–1.6	222.4	4.8	3.1–6.6	68.3	0.0	0.0–0.0	192.8	2.3	1.4–3.2	156.9	3.2	2.9–4.9	16.1
Observational study	268	0.3	0.3–0.4	27.9	2.3	1.9–2.6	38.7	0.0	0.0–0.0	89.0	1.0	0.8–1.2	32.5	2.5	2.3–2.6	10.5
Review	2098	0.4	0.3–0.4	176.5	3.3	3.1–3.5	183.1	0.0	0.0–0.0	178.0	1.4	1.3–1.5	106.6	2.9	2.7–2.9	23.2
Systematic review	231	0.7	0.6–0.9	176.5	4.1	3.6–4.4	53.6	0.0	0.0–0.0	178.0	1.9	1.6–2.1	71.5	2.9	2.7–3.0	8.7
*p* value		< 0.0001			< 0.0001			< 0.0001			< 0.0001			< 0.0001		

CI, confidence interval.

### Analysis by disease indication

The numbers of publications retained in the SPT set used for disease comparisons (i.e. with the same publication type composition for each disease indication) are shown in Table 4.

**Table 4.** Numbers of publications retained in the SPT set used for disease comparisons.

	Asthma	Migraine	MS	NSCLC	Psoriasis	Rare disease	T2D	Proportion of total
	n	n	n	n	n	n	n	%
Clinical trial	225	88	175	287	139	32	394	33%
Observational study	78	31	61	42	48	11	137	11%
Review	385	150	299	417	238	54	674	56%
Total	688	269	535	746	425	97	1205	100%

MS, multiple sclerosis; NSCLC, non-small cell lung cancer; SPT, standardised publication types; T2D, type 2 diabetes.

**Figure 4.**
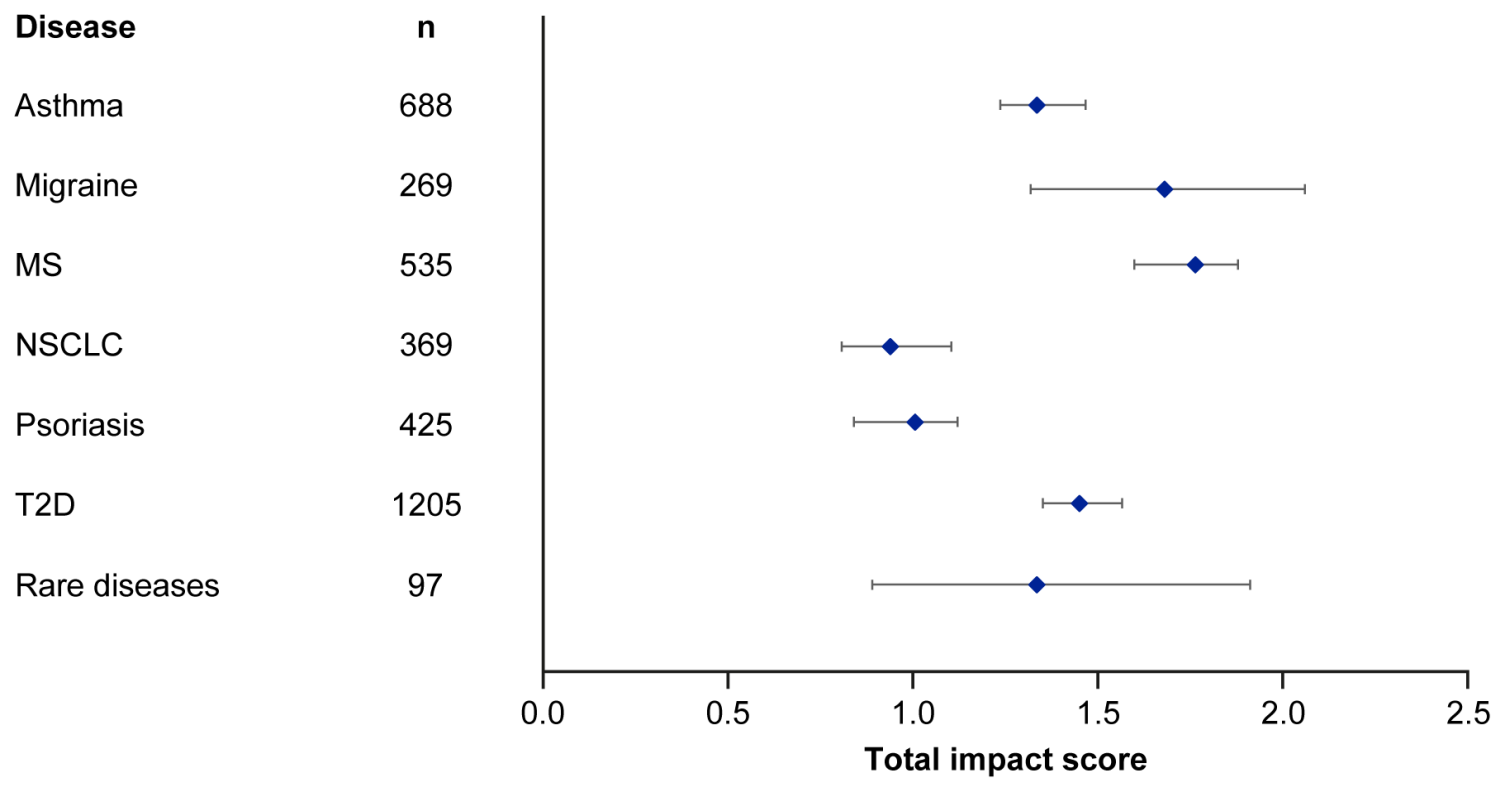
Median (95% CI) total impact scores for each disease (standardised set). MS, multiple sclerosis; NSCLC, non-small cell lung cancer; T2D, type 2 diabetes.

Median EMPIRE Index scores and journal CiteScores for each disease in the SPT set are shown in Figure 4 and Table 5. Kruskall–Wallis testing indicated at least one significant pairwise difference in the total scores, each component score and journal CiteScore. Migraine and multiple sclerosis (MS) had the highest impact across social and scholarly component scores as well as the total impact score, while NSCLC and psoriasis had the lowest. Most articles across all diseases had no societal impact, with significant differences in societal impact driven by outliers. The eight publications with the highest societal impact were all important clinical outcomes trials (three in type 2 diabetes, three in NSCLC and one each in migraine and asthma).

**Table 5.** Median and maximum scores for each EMPIRE Index component and CiteScore by disease (SPT set).

		Social			Scholarly			Societal			Total			CiteScore		
	n	Median	95% CI	Max	Median	95% CI	Max	Median	95% CI	Max	Median	95% CI	Max	Median	95% CI	Max
Asthma	688	0.6	0.4–0.7	122.5	3.0	2.8–3.2	70.5	0.0	0.0–0.0	178.0	1.3	1.2–1.5	88.0	2.9	2.7–3.0	16.1
Migraine	269	0.6	0.4–0.9	222.4	3.3	2.8–3.9	68.3	0.0	0.0–0.0	192.8	1.7	1.3–2.1	156.9	2.9	2.4–2.9	16.1
MS	535	0.9	0.7–1.0	142.1	3.8	3.4–4.2	267.4	0.0	0.0–0.0	133.5	1.8	1.6–1.9	150.0	2.8	2.6–2.9	16.1
NSCLC	369	0.1	0.1–0.1	256.1	2.4	1.9–2.8	259.7	0.0	0.0–0.0	267.0	0.9	0.8–1.1	186.7	3.2	2.9–3.6	23.2
Psoriasis	425	0.1	0.1–0.3	60.7	2.4	2.0–2.8	85.8	0.0	0.0–0.0	133.5	1.0	0.8–1.1	51.3	2.4	2.2–2.4	10.3
T2D	1205	0.4	0.3–0.4	833.4	3.4	3.1–3.7	485.5	0.0	0.0–0.0	548.8	1.5	1.4–1.6	532.5	2.8	2.8–3.0	19.1
Rare diseases	97	0.3	0.1–0.6	236.1	3.2	2.2–4.2	143.4	0.0	0.0–0.0	89.0	1.3	0.9–1.9	136.4	3.3	2.5–3.7	16.1
*p* value		< 0.0001			< 0.0001			0.0002			< 0.0001			< 0.0001		

CI, confidence interval; MS, multiple sclerosis; NSCLC, non-small cell lung cancer; T2D, type 2 diabetes.

**Figure 5.**
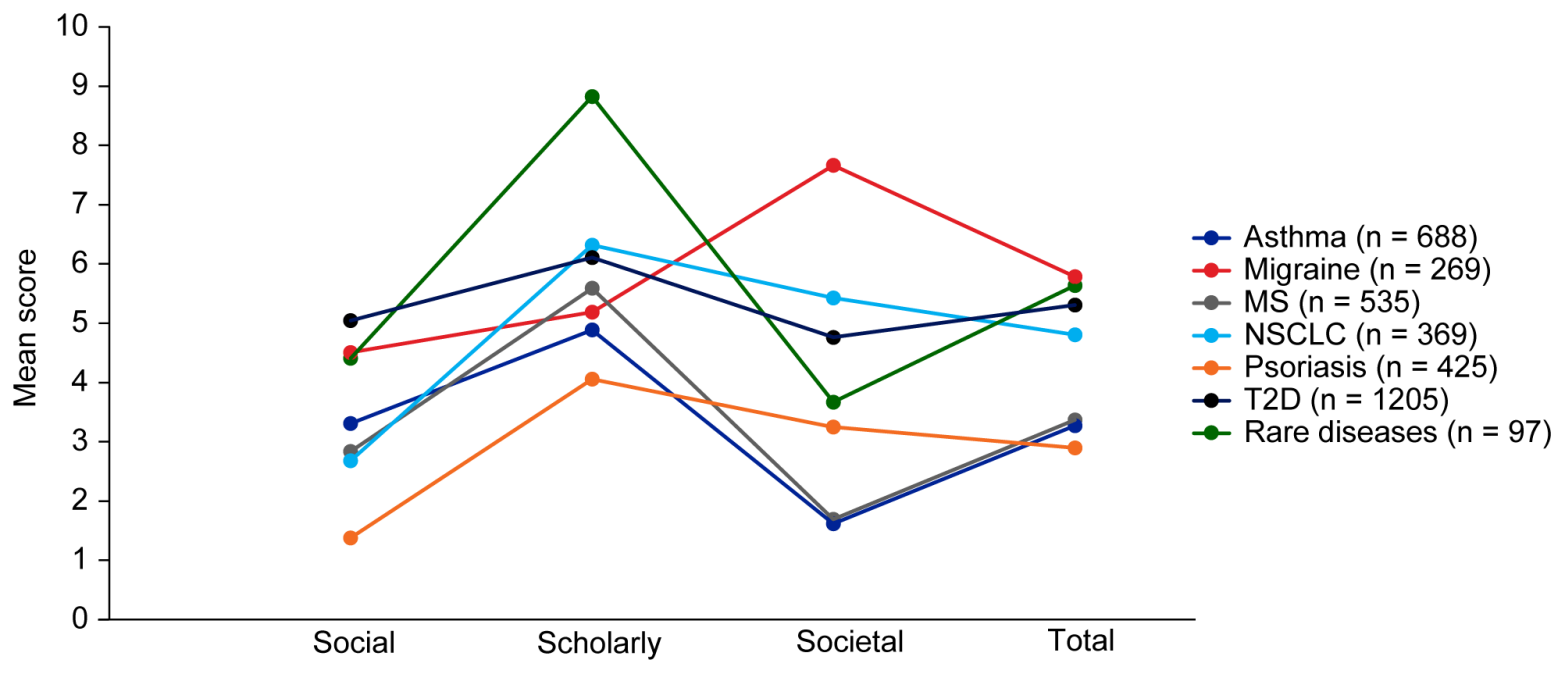
Mean EMPIRE Index scores for each disease (SPT set). The interactive version (online only, accessible here: https://s3.eu-west-2.amazonaws.com/ox.em/webflow/p29ieu21/chart2.html) also shows mean EMPIRE Index scores for each disease by publication type (full set). MS, multiple sclerosis; NSCLC, non-small cell lung cancer; T2D, type 2 diabetes.

Mean EMPIRE Index scores for each disease in the SPT set are shown in Figure 5. The interactive version of Figure 5 (online publication only) also shows the mean EMPIRE Index scores by disease for each publication type (full data set). Mean scores do not show clear trends for differences between disease indications, although societal impact appears to be lower for asthma and MS, and higher for migraine than other diseases. The high societal impact for migraine was driven by review articles; 16 of the 23 migraine articles with societal impact scores above zero were review articles. The scholarly impact for rare diseases appears to be higher than for other disease areas, albeit with low confidence owing to small numbers of publications included.

## Discussion

This analysis found that typical EMPIRE Index scores vary across both disease indications and publication types. These results provide valuable contextual information for interpreting EMPIRE Index scores and publication metric findings in general, for individual publications. For example, these findings can be used to help to understand whether a particular publication has notably high (or low) metrics.

We found considerable differences between disease areas, which broadly reflected public interest in the disease as assessed through Google search interest. Google search interest reflects the volumes of searches conducted on Google by the general public, and can be taken as an indication of the number of people actively seeking information on different topics. We found that the three diseases with the highest median EMPIRE Index scores, especially social impact, were migraine, MS and asthma; these also had the highest public interest. These differences were not observed in journal CiteScores, meaning that the disease areas with higher EMPIRE Index impact were not necessarily published in ‘high impact’ journals. NSCLC had low public interest (‘lung cancer’ as a general term was higher, but still lower than any of the other five major disease areas examined). Publications in NSCLC also had low median total impact scores, particularly in terms of social impact scores, despite being published in journals with higher median CiteScores. Overall, this suggests that the reasons that publications in some disease areas attract higher social impact scores (driven by citations in news articles and social media) is that these disease areas are of greater interest to the general public.

Although this suggests distinct differences between diseases in terms of publication impact, it should be noted that the period of interest was only a single year. The findings could therefore have been influenced by the completion of important clinical studies, which can vary from year to year across disease areas.

A clear picture is seen for publication types, with phase 3 trials demonstrating much higher metrics than other types. Phase 3 clinical trials are the last stage of clinical research of new drug treatments, and are typically large scale and provide evidence intended to guide treatment practice ^10^. The higher impact observed for this publication type likely relates to higher public interest, higher scholarly interest, and greater likelihood of citations in guidelines and policy documents. Systematic reviews had higher impact than general reviews; interestingly, this was despite being published in journals with similar median CiteScores. This likely reflects that the methodological approach to synthesising systematic literature reviews makes them more impactful. Observational studies had the lowest impact, suggesting observational analyses are still generally regarded as having lower interest.

In general, across both publication types and disease indications, median scores were higher for scholarly impact than for social or societal impact, while mean and maximal scores were broadly similar (or lower). This suggests that score distribution is more skewed for social and societal impact, with many papers generating little interest despite some scholarly impact.

A key strength of this study is the use of an automated approach to identify a large pool of publications for analysis. However, the automated process used depends on the reliability of the underlying data. For example, disease areas were identified through a PubMed search on article titles, which may have excluded some relevant articles or included irrelevant ones. The PubMed search engine uses automatic term mapping, which usually makes the search more inclusive but can introduce inconsistencies ^11^. Publication types were identified by metadata tags, but these can often be inconsistently applied or missing. It can also result in duplication; for example, some phase 3 clinical trial publications in our sample were also classified as clinical trials.

In conclusion, the EMPIRE Index successfully identified differences in impact by disease indication and publication type. This supports the notion that there is no universal gold standard metric for publications, and instead the impact of each publication needs to be evaluated in the context of the type of publication, disease area and potentially other factors. These findings should be considered when using the EMPIRE Index to assess publication impact.

## Data Availability

Figshare: EMPIRE Index disease and publication type analysis. https://doi.org/10.6084/m9.figshare.17072435.v1 ^9^ This project contains the following underlying data: SMA metrics unlinked 11Jul20.xlsx Psoriasis metrics unlinked 11Jul20.xlsx NSCLC metrics unlinked 5Jul20.xlsx NET metrics unlinked 11Jul20.xlsx NASH metrics unlinked 11Jul20.xlsx MS metrics unlinked 5Jul20.xlsx Migraine metrics unlinked 5Jul20.xlsx Google search interest (30Jul21).xlsx DLBCL metrics unlinked 11Jul20.xlsx Asthma metrics unlinked 5Jul20.xlsx TSC metrics unlinked 11Jul20.xlsx TNBC metrics unlinked 11Jul20.xlsx T2DM metrics unlinked 5Jul20.xlsx Data are available under the terms of the Creative Commons Attribution 4.0 International license (CC-BY 4.0).
