# Supplementary material for: Does the impact of medical publications vary by disease indication and publication type? An exploration using a novel, value-based, publication metric framework: the EMPIRE Index
Source: F1000Res. 2024 Oct 30;11:107. Originally published 2022 Jan 27. [Version 5] doi: 10.12688/f1000research.75805.5 (PMC11549536; doi:10.12688/f1000research.75805.5)
Supplement: Peer review history

# Reviewer report: Reviewer response for version 5

**DOI:** 10.5256/f1000research.173716.r336422

**Published:** 2024-11-08

**Authors:** Arman Yurisaldi Saleh

**Affiliations:** 1 Universitas Pembangunan Nasional Veteran Jakarta, Jakarta, Special Capital Region of Jakarta, Indonesia

The author has answered my feedback well and performed statistical regression, also explaining terms that were previously unclear.

Is the work clearly and accurately presented and does it cite the current literature?

Yes

If applicable, is the statistical analysis and its interpretation appropriate?

No

Are all the source data underlying the results available to ensure full reproducibility?

Yes

Is the study design appropriate and is the work technically sound?

Yes

Are the conclusions drawn adequately supported by the results?

Partly

Are sufficient details of methods and analysis provided to allow replication by others?

No

Reviewer Expertise:

Neurorestoration, neurobehavior, neuroprotective agent, bibliometric

I confirm that I have read this submission and believe that I have an appropriate level of expertise to confirm that it is of an acceptable scientific standard.

# Reviewer report: Reviewer response for version 4

**DOI:** 10.5256/f1000research.168629.r324069

**Published:** 2024-10-10

**Authors:** Shir Aviv-Reuven

**Affiliations:** 1 Bar Ilan University, Ramat Gan, Israel

The authors have responded to all my comments and the manuscript, I have no further comments and I now Approve it without revisions.

Is the work clearly and accurately presented and does it cite the current literature?

Partly

If applicable, is the statistical analysis and its interpretation appropriate?

I cannot comment. A qualified statistician is required.

Are all the source data underlying the results available to ensure full reproducibility?

Partly

Is the study design appropriate and is the work technically sound?

Partly

Are the conclusions drawn adequately supported by the results?

Partly

Are sufficient details of methods and analysis provided to allow replication by others?

Partly

Reviewer Expertise:

NA

I confirm that I have read this submission and believe that I have an appropriate level of expertise to confirm that it is of an acceptable scientific standard.

# Reviewer report: Reviewer response for version 4

**DOI:** 10.5256/f1000research.168629.r327843

**Published:** 2024-09-30

**Authors:** Arman Yurisaldi Saleh

**Affiliations:** 1 Universitas Pembangunan Nasional Veteran Jakarta, Jakarta, Special Capital Region of Jakarta, Indonesia

This article has strengths when viewed from the amount of data used, which is quite a lot in the form of articles, so that it provides a fairly clear picture when analyzed.

 Actually, this article would be more convincing if the right statistical test was used, but no statistical test was carried out in the manuscript.

 For example, a statistical test can be added whose results are a picture of the scientific score and social impact score for various articles in the form of systematic reviews, phase 3 clinical trials and observational.

 The difference in impact scores between the two groups can be analyzed using the t-test in statistical tests

 So this is the main suggestion for improving the article.

 In the manuscript, it can be seen that the dependent variables are, for example, the social and scientific impact scores. While the independent variables are public interest, disease indications, number of article citations, type of publication. There are shortcomings in terms of statistical analysis, related to the study of the relationship between the variables above, for example, what if you want to know the relationship between the social and scientific impact scores and public interest, and this is not found in the manuscript. A small point to note is that a linear regression analysis was not included. I kindly suggest that statistical calculations in the form of linear regression be added.

 I suggest that the author be clearer in explaining Cite Score, Altimetrik and Google Trends. A more detailed description is still needed regarding the 3 terms above so that the reader is clearer.

 My conclusion is that the author has responded well to the reviewers' input, and I provide suggestions for improvement in terms of the use of statistical tests and more detailed descriptions. The article is worthy of being indexed.

Is the work clearly and accurately presented and does it cite the current literature?

Yes

If applicable, is the statistical analysis and its interpretation appropriate?

No

Are all the source data underlying the results available to ensure full reproducibility?

Yes

Is the study design appropriate and is the work technically sound?

Yes

Are the conclusions drawn adequately supported by the results?

Partly

Are sufficient details of methods and analysis provided to allow replication by others?

No

Reviewer Expertise:

Neurorestoration, neurobehavior, neuroprotective agent, bibliometric

I confirm that I have read this submission and believe that I have an appropriate level of expertise to confirm that it is of an acceptable scientific standard, however I have significant reservations, as outlined above.

# Response

**Published:** 2024-10-18

**Authors:** Tomas Rees

**Affiliations:** Oxford PharmaGenesis, UK

We thank the reviewer for their comments. The initial version of this analysis provided only simple statistical analysis (a non-parametric test of between-group differences) because we were primarily interested in understanding the magnitude of the scores that could be expected for different kind of publications in different disease areas. Furthermore, the data are complicated to analyse because they are zero inflated (many publications have a score of 0), highly skewed (a few publications score very highly) and partially nested (some, but not all, 'phase 3 clinical trials' are also tagged as 'clinical trials', for example). 

 However, we do agree that statistical analysis can provide useful and interesting perspective on the relationship between our independent variables and the EMPIRE Index scores. Therefore, we have provided a mixed model analysis as well as a regression analysis versus Google search interest. The results of these analyses largely accord with our original conclusions, but provide rich detail on these interactions.

# Reviewer report: Reviewer response for version 3

**DOI:** 10.5256/f1000research.145010.r234323

**Published:** 2024-02-27

**Authors:** Shir Aviv-Reuven

**Affiliations:** 1 Bar Ilan University, Ramat Gan, Israel

This manuscript aims to identify impact differences between medical publications according to diseases and publication types as well as observe variations in public interest by comparing the EMPIRE index with Google search trends.

 In general I find this paper interesting, but I found it lacking in aspects which the previous reviewer mentioned. Further more, the references to related literature is lacking and there is no discussion related to other possible article level metrics and why the EMPIRE index was selected.

**Introduction**

 1. I agree with previous reviewer that Introduction is lacking, not only in presentation of the research problem, but also in the significance of this study.

 2. I further agree that Literature review section is missing. it should either be added or the introduction further expanded to address related studies utilizing this indicator as well those referring to analysis of medical publications in general and specifically those who refer to the 12 diseases under analysis in this paper.

 3. last paragraph of Introduction should be rephrased as it is unclear, particularly this - "We also look to see if there are similar variations in public interest in therapy areas we explore, with particular interest in comparing this with the EMPIRE Index includes a social score."

**Methods**

 4. The authors do not explain why the different types were selected for analysis and what each of these types presents.

 These should be detailed.

 5. The authors do not give detailed the usage of Google Trends. what data was downloaded? was it filtered to specific areas?. Further more, as some of the papers may have been published towards the end of that period, it may have been more appropriate a longer period for the trends.

**Results**

 1. The usage of CiteScore is unclear to me, as CiteScore aims to measure journal impact.

**Discussion**

 The paragraph detailing the findings related to Google trends should be moved to results and a plot showing these trends should be added.

 Discussion of the results seems short and in some cases state the obvious. for example " Observational studies had the lowest impact, suggesting observational analyses are still generally regarded as having lower interest."

 A general discussion of possible significance of the findings should be added.

Is the work clearly and accurately presented and does it cite the current literature?

Partly

If applicable, is the statistical analysis and its interpretation appropriate?

I cannot comment. A qualified statistician is required.

Are all the source data underlying the results available to ensure full reproducibility?

Partly

Is the study design appropriate and is the work technically sound?

Partly

Are the conclusions drawn adequately supported by the results?

Partly

Are sufficient details of methods and analysis provided to allow replication by others?

Partly

Reviewer Expertise:

Bibliometrics, Scientometrics, academic search, journals impact

I confirm that I have read this submission and believe that I have an appropriate level of expertise to confirm that it is of an acceptable scientific standard, however I have significant reservations, as outlined above.

# Response

**Published:** 2024-06-28

**Authors:** Tomas Rees

**Affiliations:** Oxford PharmaGenesis, UK

We thank the Dr Aviv-Reuven for their constructive comments and suggestions on our manuscript. We have revised the manuscript accordingly and addressed each comment in detail below.

**Reviewer comments and responses**

**Introduction**

 1. I agree with previous reviewer that Introduction is lacking, not only in presentation of the research problem, but also in the significance of this study.

**Response: **We have revised the introduction to provide more background on the research problem and the significance of this study. We have added a paragraph that explains the limitations of conventional metrics such as journal impact factor and CiteScore, and the advantages of using article-level metrics such as altmetrics to assess the impact of medical publications. We have also added a paragraph that describes the EMPIRE Index. We have highlighted the novelty and relevance of our study, which is the first to explore the variations in EMPIRE Index scores across different disease indications and publication types.

 2. I further agree that Literature review section is missing. it should either be added or the introduction further expanded to address related studies utilizing this indicator as well those referring to analysis of medical publications in general and specifically those who refer to the 12 diseases under analysis in this paper.

**Response:** We have added a literature review where we discuss the previous studies that have used altmetrics to assess the impact of medical publications in various fields and contexts.

 3. last paragraph of Introduction should be rephrased as it is unclear, particularly this - "We also look to see if there are similar variations in public interest in therapy areas we explore, with particular interest in comparing this with the EMPIRE Index includes a social score."

**Response:** We have rephrased the last paragraph of the introduction to clarify our research objectives and hypotheses.

**Methods**

 4. The authors do not explain why the different types were selected for analysis and what each of these types presents. These should be detailed.

**Response: **We have added a paragraph in the methods section that explains the rationale for selecting the different publication types for analysis and what each of these types represents.

 5. The authors do not give detailed the usage of Google Trends. what data was downloaded? was it filtered to specific areas?. Further more, as some of the papers may have been published towards the end of that period, it may have been more appropriate a longer period for the trends.

**Response: **We have added more details on the usage of Google Trends in the methods section.

**Results**

 The usage of CiteScore is unclear to me, as CiteScore aims to measure journal impact.

**Response:** We have clarified the rationale for the  comparison with of CiteScore in the introduction.

**Discussion**

 The paragraph detailing the findings related to Google trends should be moved to results and a plot showing these trends should be added.

**Response:** We have clarified that the data are presented in Table 1 as averages from 2019. We have not included a plot of the weekly data for 2019 as we do not refer to these and they are not relevant to our discussion. However, they are  available in the supplementary material in excel form.

 Discussion of the results seems short and in some cases state the obvious. for example " Observational studies had the lowest impact, suggesting observational analyses are still generally regarded as having lower interest."

**Response: **We have clarified the value of observational studies.

 A general discussion of possible significance of the findings should be added.

**Response: **We have added a discussion of potential significance, as well as expanding the limitations.

# Reviewer report: Reviewer response for version 2

**DOI:** 10.5256/f1000research.126135.r134659

**Published:** 2022-04-19

**Authors:** José Luis Ortega

**Affiliations:** 1 Institute for Advanced Social Studies (IESA-CSIC), Córdoba, Spain

The text is the same as the version 1. Only references were updated.

Is the work clearly and accurately presented and does it cite the current literature?

Partly

If applicable, is the statistical analysis and its interpretation appropriate?

Yes

Are all the source data underlying the results available to ensure full reproducibility?

Partly

Is the study design appropriate and is the work technically sound?

Partly

Are the conclusions drawn adequately supported by the results?

No

Are sufficient details of methods and analysis provided to allow replication by others?

No

Reviewer Expertise:

Bibliometrics, altmetrics, academic search engines, scholarly social networks

I confirm that I have read this submission and believe that I have an appropriate level of expertise to state that I do not consider it to be of an acceptable scientific standard, for reasons outlined above.

# Response

**Published:** 2023-03-10

**Authors:** Tomas Rees

**Affiliations:** Oxford PharmaGenesis, UK

We thank the reviewer for the commentary and perspective, and we have made numerous clarifications and additions to the manuscript to address these. One important caveat is that this was intended as a brief report. It follows on from the full publication detailing the background and rationale of the EMPIRE Index published in PLOS ONE, which covers how it was developed and what the component scores signify. The current report provides some useful additional investigation of the properties of the EMPIRE index and its underlying article-level metrics. It is for these reasons that this report does not include a full literature review and does not go into the details of the EMPIRE Index that are covered in the PLOS ONE paper. We originally referenced the preprint in the current submission (the primary publication had been approved but publication was delayed) but have updated the reference now that the original article is fully published.

 We have addressed the reviewer’s other comments, as follows:

 We expanded the introduction to provide greater clarity of the objectives and purpose of this analysis – including why we have used CiteScore and Google Trends. We have added information on data extraction from Altmetrics Explorer and PlumX. We have also added information on the definition of the publication types (these are defined by PubMed).

 While most of the component metrics of the EMPIRE Index are altmetrics, article citations are not and we agree that using altmetrics as an umbrella term is therefore inappropriate: we have changed to article-level metrics (ALMs) throughout.

 We agree that computing an average CiteScore would be inappropriate, because such an average would not indicate the average number of citations per paper across the set of journals and so could be misleading. However, in our analysis we use the median, which is robust in this case since it does not transform the underlying data.

 We do not believe that it is obvious whether the metrics for a given paper are notably high or low. This can only be understood by understanding the typical metrics of comparable publications, which is the objective of this report.

 We note in the discussion that the higher scores of migraine publications, in particular social scores, may be linked to higher public interest as evidence by higher Google Trends interest. However, we acknowledge that the analyses presented do not and cannot explain the differences in typical scores seen across therapy areas and publication types. Therefore, any such discussion must necessarily be speculative and as such we keep it to a minimum.

 The sole purpose of this report is to demonstrate that these variations exist and to explore the magnitude of these variations. The analyses presented are robust and do provide meaningful results that address the objective. We therefore believe that the current, amended report is worthy of publication despite its brief nature.

# Reviewer report: Reviewer response for version 1

**DOI:** 10.5256/f1000research.79727.r121444

**Published:** 2022-02-10

**Authors:** José Luis Ortega

**Affiliations:** 1 Institute for Advanced Social Studies (IESA-CSIC), Córdoba, Spain

This manuscript attempts to find impact differences between medical publications according to diseases and publication types. These differences are calculated according to a new indicator, EMPIRE Index. More than 20k publications were retrieved from PubMed about 12 diseases areas. The resulting publications were also grouped by publication type. Using the Kruskal-Wallis test, authors found that the EMPIRE Indicator produces some differences between publications according to diseases and type of documents. These results allow them to justify the utility of this indicator and its power to identify different types of impacts.

 However, the paper presents several weaknesses that impede my recommendation for approval. Next, I detail some problem points by section:

**Introduction**

 The introduction is poor because it does not introduce the research problem properly. An introduction should explain why the research is necessary and what the problem is that the paper wants to address. Why is important to know that impact varies by disease or by publication type? Why is an indicator such as EMPIRE Index necessary?

 Authors confound article-level with altmetrics. Many altmetric measures could be article-level metrics, but there are other article-level metrics (i.e. citations) that are not altmetrics. Author should revise the text and correct these statements.

 I do not see a key section in a scientific article: a literature review. Authors should include a section where they contextualize the research, explaining previous studies that had deal with this problem. Studies about new indicators, differences in the impact by subject or document type, different types of impacts, etc. Overall, authors should present other studies in the introduction that show why is important to know that a disease has more social or scholarly impact, or why some document types attract more citations or tweets. In short, more conceptual background. The paper includes only eight references, which illustrates the poor context of this research.

**Objective**

 I think this section is the most important in a paper and it is missing in this manuscript. If there are not objectives, there are not research goals and a paper lacks sense. The final line of Introduction says: “Here, we present an exploratory investigation of whether disease indications and publication types influence the average EMPIRE Index scores.” Why do you want to know this? Why is important to know this? Authors should introduce a section for objectives where they explain the main objectives and secondary ones. It could be interesting to include some research questions that specify the aim of the paper.

**Methods**

 Authors should include more detail about the data extraction process in Altmetric and PlumX. Authors claim that they used Altmetric Explorer to retrieve 20k publications. How did you exactly retrieve the data from this site? Do you use the API endpoint? How do you obtain the data from PlumX?

 A key element in the paper is to test differences according to diseases and document types, but there is not any explanation about these typologies. For example, and about document types, what is the difference between “review” and “systematic review”? This part is key because the definition of groups and types influences the results, so it is very important to define the diseases and the typology of documents.

 All the analysis relies on a new indicator, EMPIRE Index. In spite of the importance of this indicator in the paper, there is not any information about how it is calculated and conceptualized. We have to read a non-peer reviewed pre-print deposited in a repository to know what it is. I do not see this way to do science as correct. Authors have to explain how this indicator is calculated in this paper; how the three component scores are defined; what is the difference between “social impact” and “societal impact”, mainly from a conceptual point of view; likewise how do you find three components and not two or four; which metrics take part in each component and why?

 CiteScore is an indicator for journals, not for articles. How it is used and why? The same for Google Trends - how they are used in the study, and why?

**Results**

 Tables 3 and 5 use CiteScore median for valuing articles by diseases. I recall that it is not correct to use journal indicators for research articles, and the use of median or mean from a ratio (CiteScore is the ratio of citations by publications) is a mathematical artifact, and the results are spurious.

**Discussion**

 This paper lacks of any substantial discussion about the results. Sometimes the interpretation is obvious and it does not contribute valuable information. For example, the first paragraph ends: “For example, these findings can be used to help to understand whether a particular publication has notably high (or low) metrics.” This is obvious and It is not necessary to do this study to reach that conclusion. Every publication has high or low metrics. Another example: “Observational studies had the lowest impact, suggesting observational analyses are still generally regarded as having lower interest.” If they have low impact, then they have low interest.

 It is interesting that the results from the EMPIRE index are not interpreted. For example, migraine, MS and asthma are the diseases with more social impact. Why? Is this positive or negative? Is there any evidence in other studies that confirm that these pathologies are more interesting for public audiences? For example, is there any correlation between these scores and Google Trends? Is there any survey that explore the public opinion about diseases? Etc.

 This part should include the interpretation of the results according to the previous knowledge. It is interesting that this section only includes one citation (about using PubMed), but there is not any reference to previous results to discuss the validity of the findings or to explain the meaning of the results. For example, “The high impact of phase 3 clinical trials is to be expected, given that they are intended to provide practice-changing information.” Why is it that papers which provide practice-changing information should have more impact?

Is the work clearly and accurately presented and does it cite the current literature?

Partly

If applicable, is the statistical analysis and its interpretation appropriate?

Yes

Are all the source data underlying the results available to ensure full reproducibility?

Partly

Is the study design appropriate and is the work technically sound?

Partly

Are the conclusions drawn adequately supported by the results?

No

Are sufficient details of methods and analysis provided to allow replication by others?

No

Reviewer Expertise:

Bibliometrics, altmetrics, academic search engines, scholarly social networks

I confirm that I have read this submission and believe that I have an appropriate level of expertise to state that I do not consider it to be of an acceptable scientific standard, for reasons outlined above.

# Response

**Published:** 2023-02-28

**Authors:** Tomas Rees

**Affiliations:** Oxford PharmaGenesis, UK

We thank the reviewer for the commentary and perspective, and we have made numerous clarifications and additions to the manuscript to address these. One important caveat is that this is a F1000 Brief Report (https://f1000research.com/for-authors/article-guidelines/brief-report), and so is limited by the journal style template (no ‘Objectives’ section) and word count limit (2,500 words). It follows on from the full publication detailing the background and rationale of the EMPIRE Index published in PLOS ONE, which covers how it was developed and what the component scores signify. The current report is intended to provide some useful additional investigation of the properties of the EMPIRE index and its underlying article-level metrics. It is for these reasons that this report does not include a full literature review and does not go into the details of the EMPIRE Index that are covered in the PLOS ONE paper. We originally referenced the preprint in the current submission (the primary publication had been approved but publication was delayed) but have updated the reference now that the original article is fully published.

 We have addressed the reviewer’s other comments, as follows:

 We expanded the introduction to provide greater clarity of the objectives and purpose of this analysis – including why we have used CiteScore and Google Trends. We have added information on data extraction from Altmetrics Explorer and PlumX. We have also added information on the definition of the publication types (these are defined by PubMed).

 While most of the component metrics of the EMPIRE Index are altmetrics, article citations are not and we agree that using altmetrics as an umbrella term is therefore inappropriate: we have changed to article-level metrics (ALMs) throughout.

 We agree that computing an average CiteScore would be inappropriate, because such an average would not indicate the average number of citations per paper across the set of journals and so could be misleading. However, in our analysis we use the median, which is robust in this case since it does not transform the underlying data.

 We do not believe that it is obvious whether the metrics for a given paper are notably high or low. This can only be understood by understanding the typical metrics of comparable publications, which is the objective of this report.

 We note in the discussion that the higher scores of migraine publications, in particular social scores, may be linked to higher public interest as evidence by higher Google Trends interest. However, we acknowledge that the analyses presented do not and cannot explain the differences in typical scores seen across therapy areas and publication types. Therefore, any such discussion must necessarily be speculative and as such we keep it to a minimum.

 The sole purpose of this report is to demonstrate that these variations exist and to explore the magnitude of these variations. The analyses presented are robust and do provide meaningful results that address the objective. We therefore believe that the current, amended report is worthy of publication despite its brief nature.
